# The Question Box

**Published:** 1910-12-31

**Authors:** 


### THE QUESTION BOX.

### OUT-PATIENT ABUSE.

"R. G. C."—We hope to deal with the matter in an early issue.

### WHAT FLAG SHOULD A HOSPITAL FLY?

"A HOSPITAL SECRETARY" writes to the effect that his Board has recently been presented with a fine new flagstaff for his institution, and desires to have an answer to the question, "What flags may a hospital fly?"—The question is an interesting one, and we hope to deal with it at length in a future issue. We shall be glad if hospital authorities who have some rule on the subject will kindly communicate with us.

### REPAIR OF WATER BEDS.

"ECONOMY" writes as follows: Would you be so kind as to invite advice from your readers in answer to the following inquiry: I find difficulty in having water beds or rings repaired except when the damage is very small. In one case a hole into which a hand could be introduced was made at the bottom end of a water bed, and I am told the bed is past mending, although it is quite new. It seems to me that by cutting off a few inches from the length and resealing the bed should be as good as new again. Is such repair possible?—We regret that "Economy's" letter has been overlooked until now. It is quite possible to repair water beds damaged in the manner our correspondent describes, but whether such repair is worth while must depend largely on the extent of the damage and the condition of the rubber. This is the answer to the inquiry made by a large firm of rubber manufacturers. We shall be glad to have our readers' views on the matter.

